# The occurrence and risk factors of chemotherapy-induced neutropenia in patients with breast cancer not receiving primary G-CSF prophylaxis

**DOI:** 10.3332/ecancer.2023.1618

**Published:** 2023-11-02

**Authors:** Susanna Hilda Hutajulu, Siswi Oktariani, Agus Jati Sunggoro, Bagas Suryo Bintoro, Yufi Kartika Astari, Juan Adrian Wiranata, Irianiwati Widodo, Anita Ekowati, Mardiah Suci Hardianti, Kartika Widayati Taroeno-Hariadi, Johan Kurnianda, Ibnu Purwanto

**Affiliations:** 1 Division of Hematology and Medical Oncology, Department of Internal Medicine, Faculty of Medicine, Public Health and Nursing, Universitas Gadjah Mada/Dr. Sardjito General Hospital, Yogyakarta 55284, Indonesia; 2 Division of Hematology and Medical Oncology, Department of Internal Medicine, Dr. Soeradji Tirtonegoro General Hospital, Klaten 57424, Indonesia; 3 Division of Hematology and Medical Oncology, Department of Internal Medicine, Faculty of Medicine, Universitas Sebelas Maret, Surakarta 57126, Indonesia; 4 Department of Health Behavior, Environment, and Social Medicine, Faculty of Medicine, Public Health and Nursing, Universitas Gadjah Mada, Yogyakarta 55281, Indonesia; 5 Center of Health Behavior and Promotion, Faculty of Medicine, Public Health and Nursing, Universitas Gadjah Mada, Yogyakarta 55281, Indonesia; 6 Division of Hematology and Medical Oncology, Department of Internal Medicine, Dr. Sardjito General Hospital, Yogyakarta 55284, Indonesia; 7 Clinical Epidemiology Program, Master of Clinical Medicine Postgraduate Program, Faculty of Medicine, Public Health and Nursing, Universitas Gadjah Mada, Yogyakarta 55281, Indonesia; 8 Academic Hospital, Universitas Gadjah Mada, Yogyakarta 55291, Indonesia; 9 Department of Anatomical Pathology, Faculty of Medicine, Public Health and Nursing, Universitas Gadjah Mada/Dr. Sardjito General Hospital, Yogyakarta 55284, Indonesia; 10 Department of Radiology, Faculty of Medicine, Public Health and Nursing, Universitas Gadjah Mada/Dr. Sardjito General Hospital, Yogyakarta 55284, Indonesia

## Abstract

**Background:**

Chemotherapy-induced neutropenia (CIN) is a substantial side effect in chemotherapy of breast cancer patients. Administration of granulocyte colony stimulating factor (G-CSF) that may reduce CIN occurrence is not commonly available to many local cases.

**Objectives:**

To investigate the occurrence of grade 4 CIN and the influencing factors in breast cancer patients not receiving G-CSF prophylaxis.

**Methods:**

One-hundred and eighty-six newly diagnosed breast cancer patients who received a 3-weekly (neo)adjuvant or palliative chemotherapy without primary G-CSF prophylaxis were included. Grade 4 CIN was defined as absolute neutrophil count (ANC) <0.5 × 10^3^/mm^3^ during any chemotherapy cycle. We used logistic regression to explore the association of clinical, pathological and treatment factors with the risk of grade 4 CIN in the first cycle and in any given cycle.

**Results:**

Fifty-seven (30.6%) patients experienced grade 4 CIN in the first cycle and 145 (78%) had it at least once during chemotherapy. In the first cycle, haemoglobin, ANC, and albumin levels were associated with grade 4 CIN (OR = 1.48, *p* = 0.031; OR = 0.68, *p* = 0.006; and OR = 2.07, *p* = 0.042). In any cycle, pre-treatment ANC levels and anthracycline-taxane combination regimen were associated with grade 4 CIN (OR = 0.78, *p* = 0.032 and OR = 3.64, *p* = 0.012).

**Conclusions:**

A significant proportion of the local breast cancer cases undergoing chemotherapy without primary G-CSF prophylaxis experienced grade 4 CIN. Haemoglobin, ANC, and albumin levels are the risk factors for first cycle CIN, while pre-treatment ANC levels and anthracycline-taxane chemotherapy regimen are associated with CIN in any given cycle. These risk factors may be used to direct a recommendation of G-CSF prophylaxis to the most at-risk individuals in the local setting or other settings in similar situations.

## Introduction

In Indonesia, breast cancer has the highest incidence and is the second leading cause of cancer-related death. Age-standardised incidence and death rates per 100,000 women were projected to be 44 and 15.3 in 2020 [1]. In Yogyakarta Province, breast cancer cases are typically discovered in advanced stages with 5-year overall survival rate of 51% [2, 3].

Chemotherapy is one of the modalities most frequently administered to patients with breast cancer. Chemotherapy-induced neutropenia (CIN) is one of the most common side effects in breast cancer chemotherapy. Common terminology criteria for adverse events (CTCAE) grades the severity of the CIN into four grades [4]. The increased severity grades and duration of CIN predispose patients to potentially life-threatening complications, including infections and febrile neutropenia (FN). These conditions generally require hospitalisation and antibiotic treatment resulting in increased costs and unfavourable effects on patients’ quality of life. These complications, further, often lead to reduced chemotherapy doses and delays, leading to reduced relative dose intensity (RDI) and negatively impacting treatment outcomes [5].

Granulocyte colony-stimulating factors (G-CSFs) prophylaxis can significantly reduce the risk, severity, and duration of severe CIN and FN [6]. Despite these benefits, G-CSFs are not applied to all patients receiving myelosuppressive chemotherapy because of the costs associated with their routine administration. Selecting patients at greater risk for neutropenic complications may optimise their cost-effective use by directing treatment toward cases most likely to benefit from its use [7]. The European Organization for Research and Treatment of Cancer (EORTC) recommends that clinical decision-making be based on the relative myelotoxicity of patients’ treatment regimens and the existence of potential risk factors [8]. The risk factors that are predictive of the development of FN can be individual-related (older age, particularly ≥65 years old, female sex, high body surface area, poor performance status, presence of comorbidities and poor nutritional status) and disease-related (advanced disease stage, prior episode of FN and anaemia). Prophylaxis with G-CSFs is also indicated for those receiving chemotherapy with an FN risk ≥20% and those receiving chemotherapy with an FN risk of 10%–20% and present with risk factors. Further, a variability of toxicity response to standard chemotherapy exists indicating a genetic factor that may link to ethnicity [9]. Accordingly, it is essential to understand the local problem's burden and seek the risk factors for myelosuppressive toxicity that may be unique.

Capturing FN rates in real-world settings outside clinical trials is often challenging since it requires that patients consistently alert their clinicians to the development of fever and be available for absolute neutrophil count (ANC) quantification. This has made previous studies investigate the predictive utility of grade 4 [10–13]. Previous meta-analysis has revealed a significant association between grade 4 CIN and FN, duration of severe neutropenia, and the nadir ANC, all of which serve as predictors of morbidity. These findings suggest that the incidence of grade 4 CIN may serve as an additional, valid endpoint with the potential to predict clinical benefit [14].

In the literature, only few explorations conducted on the burden and predictors of CIN in Indonesian breast cancer patients. Previous studies have analysed CIN following the administration of anthracycline-based regimens, but only focused on limited clinical risk factors and had small sample size [10, 15]. No study has explored the issues in breast cancer patients from Yogyakarta province despite the fact that the region has the most cancer prevalence in Indonesia according to the current national health survey [16]. The low survival in our local breast cancer cases formerly reported might also be associated with chemotherapy toxicities including neutropenic complications [3]. As most our current patients are covered by government insurance they do not have access to primary G-CSF prophylaxis. Our study aimed to identify the occurrence of grade 4 CIN in local patients and seek the sociodemographic and clinical predictors.

## Methods

### Study participants and design

This study recruited participants who were enlisted in a prospective observational study examining chemotherapy side effects in patients with breast cancer and using a questionnaire adopted from CTCAE [4]. The registered subjects are patients who visited and had their first-line chemotherapy treatment in the Hematology and Medical Oncology Division, ‘Tulip’/Integrated Cancer Clinic, Dr. Sardjito General Hospital, Yogyakarta, Indonesia, between July 2018 and March 2022. Women over or equal to the age of 18 with histopathologically confirmed breast cancer, had Eastern Cooperative Oncology Group (ECOG) performance status ≤2 and without terminal illness or severe congestive heart failure were included. Chemotherapy was used in these cases as a neoadjuvant, adjuvant or palliative (with or without surgery) treatment. A total of 214 patients were eligible for the main study. For the present study, patients who had not received at least three cycles of chemotherapy, had not received a 3-weekly chemotherapy schedule, had not received anthracycline- or taxane-based regimen, and had no data of first cycle ANC were excluded. A total of 186 patients were finally recruited. The observation period was completed in November 2022. The study was authorised by the joint ethics committee from the Faculty of Medicine, Public Health and Nursing, Universitas Gadjah Mada (reference number: KE/FK/0417/EC/2018).

### Data collection

We gathered data on age and other clinical data on comorbidity, nutritional status (measured by body mass index (BMI)), cancer stage at diagnosis, and molecular subtypes. We also obtained pre-treatment laboratory parameters and details of chemotherapy regimen. Each chemotherapy cycle was monitored for the incidence of CIN, dose reduction, and dose delays.

#### Clinical characteristics

The clinical data consisted of age (<60 years versus ≥60 years), BMI (<23 kg/m^2^ or underweight to normal versus ≥23 kg/m^2^ or overweight to normal, referred to WHO BMI cut-off for Asian populations), presence of comorbidity (yes versus no, including diabetes mellitus, hypertension, dyslipidaemia, hyperuricaemia, gastritis, asthma, or allergy) and also particularly diabetes mellitus and hypertension presence (yes versus no). Pre-treatment laboratory parameters included haemoglobin, white blood cell (WBC) count, ANC, total lymphocyte count (TLC), absolute monocyte count (AMC), platelet count, creatinine, aspartate aminotransferase (AST), alanine aminotransferase (ALT), albumin, and vitamin D. Tumour and pathological data included cancer stage (early/stage I-II, locally advanced/stage III and metastatic/stage IV disease based on the 7th edition AJCC staging system) and molecular subtype (luminal A, luminal B including either with HER-2 negative or HER-2 positive, HER-2 enriched, and triple negative using immunohistochemical study). The treatment data consisted of chemotherapy setting (adjuvant and neoadjuvant versus palliative), first chemotherapy cycle regimen (anthracycline-containing versus taxane-containing), and overall chemotherapy regimen (anthracycline-containing only versus taxane-containing only versus anthracycline-taxane combination).

#### Chemotherapy-induced neutropenia

We collected ANC data from subjects receiving 3-weekly first-line intravenous chemotherapy. ANC was recorded every week after chemotherapy administration. CIN was recorded weekly based on the CTCAE version 4 grading system [4]. Grade 1 neutropenia was defined as ANC from the lower limit of normal to 1.5 × 10^3^/mm^3^, grade 2 was ANC from <1.5 to 1.0 × 10^3^/mm^3^, grade 3 was ANC from <1.0 to 0.5 × 10^3^/mm^3^, and grade 4 was ANC <0.5 × 10^3^/mm^3^. The highest grade of CIN among all cycles was chosen as the recorded CIN for analysis.

#### Treatment modalities

In the first cycle, patients received anthracycline-containing regimen (Doxorubicin or Epirubicin) or taxane-containing regimen (Docetaxel or Paclitaxel). Overall chemotherapy regimen was classified as anthracycline-only (Doxorubicin or Epirubicin – Cyclophospamide), taxane-only (Docetaxel or Paclitaxel – Carboplatin, Docetaxel or Paclitaxel, Docetaxel–Cyclophosphamide), and anthracycline-taxane combination (Doxorubicin or Epirubicin – Cyclophosphamide followed by Docetaxel or Paclitaxel). Patients did not receive G-CSF or antibiotics as primary or secondary prophylaxis for FN. Antibiotic treatment for managing FN is administered at the physicians’ discretion.

#### Dose reduction, dose delay and RDI

A dose reduction was defined as a clinically significant reduction in chemotherapy dose of 15% or more for at least one myelosuppressive agent in any cycle compared to the standard dose. A dose delay was defined as a delay of 7 or more days in administering at least one myelosuppressive agent (in any cycle) relative to the standard day of administration. The dosage reduction for patients receiving multi-agent chemotherapy was determined as the average percentage reductions for each agent. RDI was defined as the ratio of delivered dose intensity and standard dose intensity of the regimen based on the National Comprehensive Cancer Network (NCCN) guidelines. RDI was measured for each myelosuppressive agent throughout the chemotherapy course and then averaged across all of the myelosuppressive agents in a regimen. An RDI of less than 85% was designated as the clinically significant threshold for RDI reduction [17].

### Statistical analyses

The patient’s baseline characteristics data were presented as mean and SD for continuous data and frequency for categorical data. We presented the occurrence of grade 4 CIN at every chemotherapy cycle (cycle 1–8) and cumulatively for any cycle. We investigated dose reduction and dose delay occurrence throughout chemotherapy cycle, measured patient’s chemotherapy RDI, and calculated the frequency of low RDI.

We calculated the variance inflation factor (VIF) before conducting logistic regression. This test detects multicollinearity and signifies a correlation between the independent predictors in multivariate regression analysis. A high VIF value of ten or greater indicates high multi-collinearity redundancy among independent variables and is unacceptable [18]. Some of those variables were removed from multivariate regression analysis according to the VIF analysis results.

A bivariate logistic regression test was used to analyse clinicopathologic, laboratory, and treatment variables for grade 4 CIN at the first and any cycles. Laboratory parameters were grouped based on cut-off value generated with Liu Index method. A rounded cut-off values were chosen to maximise clinical practice application while maintaining the area under the receiver operating characteristic curve. All factors with a *p* < 0.250 in the bivariate analyses were further analysed using multivariate logistic regression. The results were internally validated by 1,000 bootstrap repetition for confidence interval generation. A *p*-value of <0.05 was considered significant. We used STATA software version 17 (Stata Corp., College Station, TX) for statistical analyses.

## Results

### Patient characteristics

From 214 eligible patients, 15 were excluded due to having less than 3 chemotherapy cycles, 2 due to not having 3-weekly chemotherapy schedule, 9 due to not receiving anthracycline- or taxane-containing regimen, and 2 due to missing data of the first cycle ANC. Finally, 186 patients were included for analysis. A total of 180 patients (96.8%) had an ECOG performance status of 0 to 1, and 6 patients (3.2%) had an ECOG performance status of 2. Baseline characteristics are presented in Table 1. The mean age of the cohort was 51.3 years old, ranging from 32 to 75 years old. More patients had BMI ≥23 kg/m^2^ (57%) and had at least one comorbidity (56%). Only 12.4% of the patients had diabetes mellitus and 22.6% had hypertension. The majority of patients (87, 46.8%) were diagnosed with stage III disease and the commonest molecular subtype was luminal B (73, 39.2%).

In terms of pre-treatment laboratory values, the patients had mean haemoglobin of 12.67 g/dL, mean WBC of 7.51 × 10^3^/mm^3^, mean ANC of 4.79 × 10^3^/mm^3^, mean TLC of 1.96 × 10^3^/mm^3^, mean AMC of 0.55 × 10^3^/mm^3^, mean platelet count of 308 × 10^3^/mm^3^, mean creatinine level of 0.76 mg/dL, mean AST level of 25.7 U/L, mean ALT level of 33.2 U/L, mean albumin level of 4.29 g/dL, and mean vitamin D level of 9.36 ng/mL. In the first cycle of chemotherapy, 156 (83.9%) patients received an anthracycline-containing regimen and 30 (16.1%) patients received a taxane-containing regimen. In the cumulative cycle, 24 (12.9%) patients received an anthracycline-only regimen, 26 (13.9%) patients received a taxane-only regimen, and 136 (73.2%) patients received an anthracycline-taxane combination. The most common chemotherapy regimen was Doxorubicin-Cyclophosphamide followed by

Docetaxel (AC-D), administered to 77 patients (41.1%). The median number of chemotherapy cycles was 8 cycles. Overall, 1,302 chemotherapy cycles were observed and after adjusting for missing data in 6 cycles, a total of 1,296 chemotherapy cycles were included in the analysis.

### Dose reduction, dose delay, and RDI

More than 15% dose reduction of any chemotherapy agent has only occurred in 7 (3.8%) patients, while dose delay for more than 7 days at any cycle was found in 85 (45.7%) patients. The mean chemotherapy RDI of these patients was 92.9%. Twenty-three (12.4%) patients received less than 85% RDI.

### Occurrence of grade 4 CIN

There are 389 grade 4 CIN events (30%) observed. As shown in Table 2, the occurrence of grade 4 CIN was similar in any chemotherapy cycle. Grade 4 CIN at the first chemotherapy cycle was found in 57 events (30.6%) of patients. The highest proportion of grade 4 CIN events was observed in the seventh cycle (37.3%) and the lowest in the second cycle (22.6%). The overall grade 4 CIN incidence at any chemotherapy cycle was 78%. The observed objective FN incidence in these patients at any cycle was 10.2% (Table 2). In addition, there were 39 (21.0%) concurrences of grade 4 CIN and subjective fever symptoms reported by patients at any chemotherapy cycle resulting to a total FN occurrence of 31.2%.

### Risk factors of grade 4 CIN

Before proceeding the multivariate logistic regression analysis, VIF analysis was performed. The results are summarised in Supplementary Table 1. Even though WBC and ANC showed significant results in the bivariate logistic regression analysis, we detected strong multicollinearity between ANC (VIF = 29.52, tolerance = 0.034) and WBC values (VIF = 37.56, tolerance = 0.027). VIF analysis underscored that WBC exhibits a higher VIF than ANC, indicating a more substantial contribution to the overall multicollinearity. We excluded the pre-treatment WBC value in the regression analysis based on these results.

In the multivariate model with bootstrap validation, it is shown that pre-treatment ANC was associated with reduced risk of grade 4 CIN (OR = 0.36, 95% CI 0.15–0.84, *p* = 0.019), while haemoglobin and TLC level associated with the risk of grade 4 CIN in the first cycle of chemotherapy (OR = 2.36, 95% CI 1.05–5.31, *p* = 0.037; OR = 2.82, 95% CI 1.09–7.33, *p* = 0.033; respectively) (Table 3). For the analysis of grade 4 CIN at any chemotherapy cycle, it is shown that higher pre-treatment albumin levels are significantly associated with a higher risk of grade 4 CIN at any cycle (OR = 2.74, 95% CI 1.03–7.27, *p* = 0.044). Compared to the anthracycline-only regimen, the anthracycline and taxane combination regimen was associated with a higher risk of grade 4 CIN (OR = 3.99, 95% CI 1.09–14.59, *p* = 0.036) (Table 4).

## Discussion

Chemotherapy is the most frequent modality in breast cancer management in Yogyakarta, Indonesia, as most of patients presented in advanced stages [2]. Being widely applied, the knowledge and understanding of its toxicities will help the local cancer care delivery and management. Little is known, however, about the incidence and determinants of neutropenia in Indonesian breast cancer cases, including in our region, particularly in those not receiving primary G-CSF prophylaxis. In the present study we included cases with all stages and receiving chemotherapy either for (neo)adjuvant or palliative intention. Grade 4 neutropenia occurred frequently in the first cycle and in any chemotherapy cycles (30.6% and 78%, respectively). As to clinical and treatment-associated factors, we found that pre-treatment hemoglobin, ANC, and TLC levels significantly influenced grade 4 CIN in the first cycle. An albumin level >4.4 g/dL and anthracycline-taxane combination regimen were associated with an increased risk of grade 4 CIN throughout chemotherapy.

In our study, the incidence of grade 4 CIN in the first cycle was comparable with a previous study in Indonesia without G-CSF prophylaxis (30.6% versus 27.4%) [15], although we included all stage I–IV patients and not only those receiving adjuvant chemotherapy as it did. The grade 4 CIN incidence in our study is higher compared to similar studies that included all disease stages without primary prophylaxis in other countries such as Ethiopia (2.1%) [11], and Switzerland (18%) [19]. The incidence of grade 4 CIN in any chemotherapy cycle in our study was also higher compared to similar studies without primary G-CSF administration in Japan (32%) [20], Brazil (12.1%) [21] and Belgium (44%) [13].

When compared to a study from South Korea, our incidence of grade 4 CIN in the first cycle is much lower (30.6% versus 69.7%) [22]. The main difference between our study and Min *et al* [22] is that we included patients with stage 4 disease that comprised 20.4% of all subjects, expected to have a higher incidence of grade 4 CIN. The study by Min *et al* [22] only includes patients with an anthracycline-based regimen that is far more toxic, while in ours only 83.9% received anthracycline-containing regimen in the first cycle. As for the cumulative chemotherapy regimen, 73.2% of our cases received an anthracycline-taxane combination and 12.9% received anthracycline-only regimen, with a comparable incidence of grade 4 CIN in any cycle to that reported by Min *et al* [22] (78% versus 71.2%).

All differences we observed from other studies in other parts of the world indicated a genetic predisposition of CIN across populations. A more recent report demonstrated that Asian patients had significantly more grades 3–4 CIN (30.4%) compared to Caucasian patients (4%) during four cycles of docetaxel and cyclophosphamide [9]. This difference is suggested to be caused by substantial interethnic and genetic variability of chemotherapy metabolism [24]. Therefore, we surmise that the higher incidence of grade 4 CIN in our study population might also be influenced by ethnic differences, which is a future direction of our current study.

The incidence of FN in this study (10.2%) is lower compared to similar studies without primary G-CSF prophylaxis from South Korea (27.2%) [22], Japan (45.0%) [25], and Singapore (13.8%) [26]. Some of our patients reported fever a few days preceding the scheduled follow-up appointments, coupled with laboratory findings of grade 4 neutropenia. In these observations, we assumed the possibility that these patients might be experiencing FN. When observing the combined incidence of subjectively and objectively reported FN, the rate of FN in this study (31.2%) is lower than the reported incidence from the study mentioned earlier from Japan and higher than the South Korea and Singapore study. Further investigations are needed to confirm these findings, as the incidence of combined FN is not solely measured with objective temperature readings.

Our findings demonstrated that anthracycline-taxane combination significantly increase the risk of grade 4 CIN at any cycle, which is supporting previous studies [27, 28] and existing chemotherapy regimen classification according to the EORTC [8]. In addition to this expected risk factor, we found that pre-treatment ANC >4 × 10^3^/mm^3^ was a protective factor for grade 4 CIN in the first cycle of chemotherapy, which is in line with other studies [11]. Higher pre-treatment ANC may indicate higher bone marrow reserve. After receiving myelosuppressive chemotherapy, patients with lower pre-treatment ANC are more likely to develop neutropenia. A previous study also found that the depth of ANC value in the first cycle predicts subsequent cycles of neutropenia [29]. Interestingly, another Indonesian study showed age, blood pressure, nutrition status and hemoglobin level as risk factors for CIN [15]. Unlike ours, Keswara *et al* [15] only focused on the four clinical risk factors mentioned. Keswara *et al* [15] also only included locally advanced breast cancer cases receiving anthracycline-containing regimens and considered all grades of CIN. Our findings may further underpin the importance of evaluating pre-treatment ANC for predicting grade 4 CIN and for considering G-CSF prophylaxis use, especially in resource-limited settings. Particularly when considering the anthracycline-taxane combination as the most frequently used regimen, which has the highest risk of FN compared to anthracycline-based and taxane-based alone, the use of G-CSF is further recommended [8].

Notably, our study found that higher pre-treatment hemoglobin, TLC, and albumin levels were associated with a higher risk of developing grade 4 CIN. Our findings about hemoglobin and TLC contradict to previous reports [15, 27], while the relationship of albumin level with CIN did not support others’ observation [12, 25]. We so far do not have any explanation for that. However, some studies in various type of cancer observed that higher hemoglobin and albumin levels, in line with the incidence of CIN in the first cycle, were significantly associated with better overall survival and disease-free survival [30–32], and offers insights into patient's nutritional status [33]. Further studies are needed to confirm the relationship between both variables and an increased risk of grade 4 CIN in the first cycle and the survival outcomes.

Our study has important clinical implications. We, align with other [15], demonstrated a high incidence of grade 4 CIN in Indonesia compared to those from other countries. More than half of our patients in this study received anthracycline-taxane combination regimen, a high-risk chemotherapy regimen for FN according to EORTC [8] and are justified to receive a G-CSF prophylaxis. The implementation of G-CSF prophylaxis has not only demonstrated a reduction in the incidence of FN-related dose delays and reductions [34], but also a decrease in infection-related and early mortality [35], although its implication on overall survival and progression-free survival requires further investigation [34,36]. However, the national healthcare insurance coverage is yet to cover the use of G-CSF prophylaxis. Thus, identification of clinical risk factors of grade 4 CIN, such as pre-treatment hemoglobin >13 g/dL, ANC >4 × 10^3^/mm^3^, and albumin >4.4 g/dL, and anthracycline-taxane combination regimen, may help choose a patient at the highest risk for developing grade 4 CIN for the effective and efficient use of G-CSFs. The further direction of our study is to investigate the role of grade 4 CIN in predicting the survival of patients with breast cancer receiving chemotherapy.

The strength of our study is that it specifically analysed grade 4 CIN predictors with data obtained in a prospective manner. We showed a good follow-up period of up to 6 months, which depicts the highest number of patients still undergoing chemotherapy. Besides, we also included pre-treatment vitamin D level in the analysis which is rarely explored, although its significant impact was not observed. We also tried to be clinically relevant by providing rounded cut-off for pre-treatment laboratory parameters, an effort to make the findings more practical for clinical use. Limitations of our study include study setting of single-centre, so that a careful consideration is required to interpret the results. Even though we already conducted bootstrap validation, it yielded unique findings from hemoglobin, TLC, and albumin factors. Another limitation of this study is the difference between the neoadjuvant or adjuvant and palliative treatment for breast cancer that might affect the incidence and factors related to grade 4 CIN. Due to the limited cohort size of patients receiving palliative treatment (38 patients, 20.4%), conducting a separate analysis could compromise comparability with the larger cohort receiving (neo)adjuvant treatments. However, it is notable that all patients included in this cohort exhibited favourable performance status (ECOG 0 to 2), with 96.8% having ECOG performance status of 0 to 1 and received either an anthracycline-based, taxane-based, or anthracycline-taxane combination regimen. We also adjusted the chemotherapy intention variable, which yielded no statistically significant result. Moreover, similar prior studies [11–13, 23, 27, 28] have also incorporated various stages of breast cancer patients in their cohort. We also did not explore the risk factors for FN in our study due to the constraints on timely and measured reporting of fever development which led to under-reporting, a challenge that was encountered by previous real-world observational studies as well [10–13]. In our setting routine clinical assessment after chemotherapy is customarily set every 1 week. When a patient suffered from a fever that might be related to FN before the appointed schedule, she might stay at home or go to other hospital so that we missed the temperature data. Although we detected patient’s report during CTCAE questionnaire on fever symptom at home, patients might not record the temperature. We identified FN only when a patient had a fever during her clinical visit with objective temperature documentation.

## Conclusion

The first cycle and any cycle of grade 4 CIN occurred in a high proportion of our patients with local breast cancer not receiving primary G-CSF prophylaxis. Local risk factors for first cycle grade 4 CIN included pre-treatment hemoglobin, ANC, and TLC level, while for any cycle grade 4 CIN included pre-treatment albumin level and combination regimen of anthracycline and taxane. The identified risk factors may provide insights into determining patients at risk for grade 4 neutropenia and consequently enable early recognition of patients who may have a higher risk of developing FN. The present data regarding the estimated incidence of FN and high use of a high-risk chemotherapy regimen for FN in our local population may also serve as a basis for recommending G-CSF prophylaxis, allowing more efficient use of medical drugs and keeping all patients on the optimal chemotherapy schedule. This is conceivably valuable for high-level authorities to call for action in improvement of breast cancer care delivery.

## Conflicts of interest

The authors declare no conflict of interest for this manuscript.

## Funding

This work was supported by The Indonesian Ministry of Research, Technology, and Higher Education (2018, grant number: 1820/UN1/DITLIT/DIT-LIT/LT-2018 and 2020, grant number: 2258/UN1/DITLIT/DIT-LIT/PT/2020).

## Figures and Tables

**Table 1.** Subject demographic and clinical characteristics (*n* = 186).

Variables	Frequency n (%)/mean ± SD
Age (years)	51.30 ± 9.26
<60 years	156 (83.9)
≥60 years	30 (16.1)
BMI (WHO Asia-Pacific)	24.03 ± 4.56
<23 kg/m^2^	80 (43.0)
≥23 kg/^m^2	106 (57.0)
Comorbidity presence	
No	82 (44.1)
Yes	104 (55.9)
Diabetes mellitus comorbidity	
No	163 (87.6)
Yes	23 (12.4)
Hypertension comorbidity	
No	144 (77.4)
Yes	42 (22.6)
Stage	
I–II	61 (32.8)
III	87 (46.8)
IV	38 (20.4)
Molecular subtypes	
Luminal A	44 (23.7)
Luminal B	73 (39.2)
HER2-enriched	35 (18.8)
TNBC	34 (18.3)
Chemotherapy intention	
Adjuvant or neoadjuvant	148 (79.6)
Palliative	38 (20.4)
Chemotherapy regimen in the first cycle	
Anthracycline-containing	156 (83.9)
Taxane-containing	30 (16.1)
Chemotherapy regimen in cumulative cycle	
Anthracycline-based	24 (12.9)
Taxane-based	26 (13.9)
Anthracycline-taxane combination	136 (73.2)
Baseline laboratory values	
Hb (g/dL)	12.67 ± 1.26
WBC (×10^3^/mm^3^)	7.51 ± 2.34
ANC (×10^3^/mm^3^)	4.79 ± 1.99
TLC (×10^3^/mm^3^)	1.96 ± 0.73
AMC (×10^3^/mm^3^)	0.55 ± 0.52
PLT (×10^3^/mm^3^)	308.41 ± 82.15
Creatinine (mg/dL)	0.76 ± 0.16
AST (U/L)	25.74 ± 34.02
ALT (U/L)	33.15 ± 67.20
Albumin (g/dL) (*n* = 176)	4.29 ± 0.58
Vitamin D (ng/mL) (*n* = 170)	9.36 ± 4.23

**Abbreviations:** SD, Standard Deviation; BMI, Body Mass Index; ECOG, Eastern Cooperative Oncology Group; TNBC, Triple Negative Breast Cancer; Hb, Hemoglobin; WBC, White Blood Cell; ANC, Absolute Neutrophil Count; TLC, Total Leukocyte Count; AMC, Absolute Monocyte Count; PLT, Platelet; AST, Serum Glutamic Oxaloacetic Transaminase; ALT, Serum Glutamic Pyruvic Transaminase

**Table 2.** Occurrence rate of grade 4 CIN, febrile neutropenia, dose reduction, and dose delay.

Variable	Chemotherapy cycles								Any cycle n (%)
	Cycle 1 *n* (%)	Cycle 2 *n* (%)	Cycle 3 *n* (%)	Cycle 4 *n* (%)	Cycle 5 *n* (%)	Cycle 6 *n* (%)	Cycle 7 *n* (%)	Cycle 8 *n* (%)	
***N***	**186**	**186**	**186**	**181**	**166**	**161**	**118**	**114**	**186**
Grade 4 CIN	57 (30.6)	42 (22.6)	47 (25.4)	56 (30.9)	44 (26.7)	57 (35.4)	44 (37.3)	42 (36.8)	145 (78.0)
Febrile neutropenia*	5 (2.7)	4 (2.2)	2 (1.1)	1 (0.6)	4 (2.4)	5 (3.1)	3 (2.5)	2 (1.8)	19 (10.2)
Dose reduction	2 (1.1)	2 (1.1)	2 (1.1)	2 (1.2)	2 (1.2)	5 (3.1)	4 (3.4)	4 (3.5)	7 (3.8)
Dose delay	-	16 (8.6)	16 (8.6)	20 (11.0)	23 (13.9)	16 (9.9)	4 (3.4)	6 (5.3)	85 (45.7)

* Absolute Neutrophil Count (ANC) <0.5 × 10^3^/mm^3^ and temperature ≥38.3°C

**Abbreviations:** CIN, Chemotherapy-induced Neutropenia

**Table 3.** Clinicopathologic-, laboratory-, and treatment-related factors associated with occurrence of grade 4 CIN in the first cycle of chemotherapy (*n* = 186).

Variable	Grade 4 CIN in first cycle (%)	Crude OR (95% CI)	*p*	Adjusted OR (95% CI)	*p*
Age					
<60 years	91.2	Ref		Ref	
≥60 years	8.8	0.40 (0.14–1.11)	0.077	0.30 (0.09–1.03)	0.056
BMI					
<23 kg/m^2^	45.6	Ref			
≥23 kg/m^2^	54.4	0.86 (0.46–1.61)	0.634		
Hemoglobin					
≤13 g/dL	45.6	Ref		Ref	
>13 g/dL	54.4	2.19 (1.16–4.15)	0.015	2.36 (1.05–5.31)	0.037*
WBC					
≤7 × 10^3^/mm^3^	61.4	Ref			
>7 × 10^3^/mm^3^	38.6	0.46 (0.24–0.87)	0.017		
ANC					
≤4 ×10^3^/mm^3^	52.6	Ref		Ref	
>4 × 10^3^/mm^3^	47.4	0.47 (0.25–0.89)	0.020	0.36 (0.15–0.84)	0.019*
TLC					
≤2.5 × 10^3^/mm^3^	70.2	Ref		Ref	
>2.5 × 10^3^/mm^3^	29.8	2.05 (0.98–4.25)	0.054	2.82 (1.09–7.33)	0.033*
AMC					
≤0.4 × 10^3^/mm^3^	22.8	Ref			
>0.4 × 10^3^/mm^3^	77.2	1.22 (0.59–2.55)	0.588		
PLT					
≤300 × 10^3^/mm^3^	61.4	Ref		Ref	
>300 × 10^3^/mm^3^	38.6	0.50 (0.27–0.95)	0.035	0.53 (0.23–1.24)	0.144
AST					
≤20 U/L	61.4	Ref			
>20 U/L	38.6	0.83 (0.44–1.58)	0.578		
ALT					
≤25 U/L	61.4	Ref			
>25 U/L	38.6	0.86 (0.45–1.63)	0.647		
Albumin					
≤4.4 g/dL	39.6	Ref		Ref	
>4.4 g/dL	60.4	1.76 (0.92–3.39)	0.089	1.66 (0.74–3.73)	0.219
Vitamin D					
≤8 ng/mL	37.2	Ref			
>8 ng/mL	62.8	1.18 (0.60–2.31)	0.633		
Comorbidity presence					
No	50.9	Ref		Ref	
Yes	49.1	0.67 (0.36–1.26)	0.216	0.65 (0.29–1.41)	0.275
Diabetes mellitus comorbidity					
No	91.2	Ref			
Yes	8.8	0.59 (0.21–1.68)	0.327		
Hypertension comorbidity					
No	75.4	Ref			
Yes	24.6	1.17 (0.56–2.45)	0.668		
Stage					
IV	19.3	Ref			
I–II	38.6	1.38 (0.58–3.32)	0.466		
III	42.1	0.94 (0.40–2.17)	0.876		
Molecular subtype					
TNBC	17.6	Ref			
Luminal A	29.8	1.51 (0.58–3.93)	0.397		
Luminal B	36.8	0.97 (0.39–2.37)	0.945		
HER2-enriched	15.8	0.83 (0.29–2.39)	0.731		
Chemotherapy intention					
Adjuvant-neoadjuvant	80.7	Ref			
Palliative	19.3	0.90 (0.41–1.98)	0.799		
Chemotherapy regimen in the first cycle					
Anthracycline-containing	82.5	Ref			
Taxane-containing	17.5	1.16 (0.50–2.66)	0.727		

* statistically significant

**Abbreviations:** CIN, Chemotherapy-induced Neutropenia; OR, Odds Ratio; CI, Confidence Interval; Ref, Reference; Cont., Continuous Data; WBC, White Blood Cell; ANC, Absolute Neutrophil Count; TLC, Total Leukocyte Count; AMC, Absolute Monocyte Count; PLT, Platelet; AST, Serum Glutamic Oxaloacetic Transaminase; ALT, Serum Glutamic Pyruvic Transaminase; TNBC, Triple Negative Breast Cancer

**Table 4.** Clinicopathologic-, laboratory-, and treatment-related factors associated with occurence of grade 4 CIN in any cycle of chemotherapy (*n* = 186).

Variable	Grade 4 CIN in any cycle (%)	Crude OR (95% CI)	*p*	Adjusted OR (95% CI)	*p*
Age					
<60 years	86.2	Ref		Ref	
≥60 years	13.8	0.49 (0.21–1.17)	0.108	0.47 (0.15–1.52)	0.208
BMI					
<23 kg/m^2^	43.4	Ref			
≥23 kg/m^2^	56.6	0.92 (0.46–1.86)	0.821		
Hemoglobin					
≤13 g/dL	58.6	Ref			
>13 g/dL	41.4	1.06 (0.52–2.16)	0.875		
WBC					
≤7 × 10^3^/mm^3^	49.0	Ref			
>7 × 10^3^/mm^3^	51.0	0.85 (0.42–1.72)	0.657		
ANC					
≤4 × 10^3^/mm^3^	44.8	Ref		Ref	
>4 × 10^3^/mm^3^	55.2	0.36 (0.16–0.80)	0.013	0.36 (0.12–1.07)	0.067
TLC					
≤2.5 × 10^3^/mm^3^	77.9	Ref			
>2.5 × 10^3^/mm^3^	22.1	1.33 (0.54–3.30)	0.532		
AMC					
≤0.4 × 10^3^/mm^3^	24.8	Ref			
>0.4 × 10^3^/mm^3^	75.2	1.15 (0.52–2.53)	0.731		
PLT					
≤300 × 10^3^/mm^3^	53.8	Ref		Ref	
>300 × 10^3^/mm^3^	46.2	0.46 (0.22–0.96)	0.038	0.64 (0.23–1.84)	0.412
AST					
≤20 U/L	58.6	Ref			
>20 U/L	41.4	0.95 (0.47–1.94)	0.899		
ALT					
≤25 U/L	57.2	Ref			
>25 U/L	42.8	1.39 (0.67–1.87)	0.378		
Albumin					
≤4.4 g/dL	43.4	Ref		Ref	
>4.4 g/dL	56.6	3.05 (1.43–6.49)	0.004	2.74 (1.03–7.27)	0.044*
Vitamin D					
≤8 ng/mL	43.5	Ref		Ref	
>8 ng/mL	56.5	0.51 (0.23–1.11)	0.090	0.58 (0.20–1.66)	0.311
Comorbidity presence					
No	43.4	Ref			
Yes	56.6	1.12 (0.56–2.25)	0.742		
Diabetes mellitus comorbidity					
No	88.3	Ref			
Yes	11.7	0.77 (0.28–2.11)	0.618		
Hypertension comorbidity					
No	75.2	Ref		Ref	
Yes	24.8	1.92 (0.75–4.95)	0.174	1.96 (0.49–7.71)	0.337
Stage					
IV	19.3	Ref			
I–II	33.8	1.46 (0.56–3.81)	0.441		
III	46.9	1.28 (0.53–3.09)	0.586		
Molecular subtype					
TNBC	20.0	Ref		Ref	
Luminal A	24.1	0.67 (0.20–2.22)	0.513	0.62 (0.08–4.87)	0.651
Luminal B	39.3	0.61 (0.20–1.84)	0.385	0.62 (0.08–4.63)	0.638
HER2-enriched	16.6	0.38 (0.11–1.23)	0.107	0.35 (0.04–3.34)	0.366
Chemotherapy intention					
Adjuvant-neoadjuvant	80.7	Ref			
Palliative	19.3	0.74 (0.33–1.69)	0.477		
Chemotherapy regimen in the cumulative cycle					
Anthracycline-based	8.3	Ref		Ref	
Taxane-based	12.4	2.25 (0.71–7.14)	0.169	1.51 (0.22–10.25)	0.674
Anthracycline-Taxane combination	79.3	5.48 (2.17–13.82)	<0.001	3.99 (1.09–14.59)	0.036*

* statistically significant

**Abbreviations:** CIN, Chemotherapy-induced Neutropenia; OR, Odds Ratio; CI, Confidence Interval; Ref, Reference; Cont., Continuous Data; WBC, White Blood Cell; ANC, Absolute Neutrophil Count; TLC, Total Leukocyte Count; AMC, Absolute Monocyte Count; PLT, Platelet; AST, Serum Glutamic Oxaloacetic Transaminase; ALT, Serum Glutamic Pyruvic Transaminase; TNBC, Triple Negative Breast Cancer

**Supplementary Table 1.** Multicollinearity test for laboratory factors of grade 4 CIN.

Variables	Before variables exclusion		After variables exclusion	
	VIF	Tolerance	VIF	Tolerance
Hb (g/dL)	1.20	0.833	1.13	0.883
WBC (×10^3^/mm^3^)	37.56*	0.027	-	-
ANC (×10^3^/mm^3^)	29.52	0.034	1.21	0.826
TLC (×10^3^/mm^3^)	5.62	0.178	1.13	0.883
AMC (×10^3^/mm^3^)	1.22	0.818	1.14	0.874
PLT (×10^3^/mm^3^)	1.28	0.780	1.20	0.830
AST (U/L)	12.17*	0.082	-	-
ALT (U/L)	11.90	0.084	1.03	0.968
Albumin (g/dL)	1.20	0.833	1.11	0.901
Vitamin D (ng/mL)	1.03	0.969	1.01	0.987

* High multicollinearity, excluded in the multivariate analysis

**Abbreviations:** CIN, Chemotherapy-induced Neutropenia; VIF, Variance Inflation Factors; Hb, Hemoglobin; WBC, White Blood Cell; ANC, Absolute Neutrophil Count; TLC, Total Leukocyte Count; AMC, Absolute Monocyte Count; PLT, Platelet; AST, Serum Glutamic Oxaloacetic Transaminase; ALT, Serum Glutamic Pyruvic Transaminase.
